# Regulation of the Host Antiviral State by Intercellular Communications

**DOI:** 10.3390/v7082840

**Published:** 2015-08-19

**Authors:** Sonia Assil, Brian Webster, Marlène Dreux

**Affiliations:** CIRI, Université de Lyon, Inserm, U1111, Ecole Normale Supérieure de Lyon, Université Lyon 1, CNRS, UMR5308, LabEx Ecofect, Université de Lyon, Lyon F-69007, France; E-Mail: sonia.assil@ens-lyon.fr

**Keywords:** virus, infection, innate immunity, interferon, inflammation, Toll-like receptor, extracellular vesicles, exosome, immature virus, cell-to-cell transmission, cell-to-cell contact

## Abstract

Viruses usually induce a profound remodeling of host cells, including the usurpation of host machinery to support their replication and production of virions to invade new cells. Nonetheless, recognition of viruses by the host often triggers innate immune signaling, preventing viral spread and modulating the function of immune cells. It conventionally occurs through production of antiviral factors and cytokines by infected cells. Virtually all viruses have evolved mechanisms to blunt such responses. Importantly, it is becoming increasingly recognized that infected cells also transmit signals to regulate innate immunity in uninfected neighboring cells. These alternative pathways are notably mediated by vesicular secretion of various virus- and host-derived products (miRNAs, RNAs, and proteins) and non-infectious viral particles. In this review, we focus on these newly-described modes of cell-to-cell communications and their impact on neighboring cell functions. The reception of these signals can have anti- and pro-viral impacts, as well as more complex effects in the host such as oncogenesis and inflammation. Therefore, these “broadcasting” functions, which might be tuned by an arms race involving selective evolution driven by either the host or the virus, constitute novel and original regulations of viral infection, either highly localized or systemic.

## 1. Introduction

Intercellular transfer of materials to neighboring cells by extracellular vesicles is increasingly recognized as an important mean of communication between cells [1]. Indeed, many cell types release various membrane-enclosed microvesicles. Such vesicles are generated from the cell surface (e.g., shedding microvesicles or CD133^+^ membrane particles), from internal compartments, including the endosome-derived membranes (e.g., exosomes and exosome-like vesicles), or upon specific activation (e.g., apoptotic bodies) [1,2]. In the context of viral infections, recent evidence has highlighted the importance of such communication in regulating virus spread and pathogenesis. This cell-to-cell communication may directly activate host responses via the transfer and recognition of viral components or indirectly by modulation of the host’s innate and adaptive responses and homeostasis, such as wound healing [3], via the transfer of host elements.

The best-characterized subclass of extracellular vesicles is the exosomes, defined as 40–100 nm-sized vesicles. Exosomes carry lipids, RNAs, and proteins from the originating cell [1,4,5]. These vesicles are likely formed in multivesicular bodies (MVBs), a subcellular compartment derived from endosomes, by the inward budding of the endosomal membrane [1]. The ESCRT machinery promotes these invaginations of intraluminal vesicles [6]. Hence, the ESCRT machinery is pivotal for the biogenesis of exosomes [7]. Additionally, this machinery is also involved in remodeling of the plasma membrane (e.g., for repair of the membrane) [8,9], implying a possible contribution to the release of other types of vesicles. The intraluminal vesicles formed within MVBs can then follow either the secretory or the lysosomal pathway. In the secretory pathway, MVBs fuse with the plasma membrane, which results in the release of intraluminal vesicles as exosomes and the incorporation of the peripheral membrane of MVBs into the plasma membrane [1,10], yet the release mechanism is still incompletely defined. Consistent with this route of biogenesis, exosomes contain components from specific cell compartments, including the cytosol, plasma membrane, and endosomes, while mitochondrial, nuclear, endoplasmic reticulum, and Golgi components are rarely found in exosomes [11]. Exosomes are produced from a broad array of cell types [11], likely nearly all cell types. As a consequence, extracellular vesicles are found in the plasma and other body fluids, including breast milk, semen, saliva, urine, and sputum [12]. The functions of exosomes in non-pathogenic conditions, defined by both *in vitro* and *in vivo* studies, have been recently well-reviewed [1,13]. Exosomes serve important functions in cell-to-cell communications through the transfer of cellular components, including diverse RNA species and proteins between cells. In accordance, several recent studies illustrate that exosomal transfer is pivotal in regulating numerous host responses. These functions vary widely depending on the context and/or system being studied. Importantly, recent research has focused on how vesicular transfer is implicated in the regulation of a broad array of viral infections. Therefore, this pathway likely regulates the progression of the infection and pathogenesis, although in some contexts, the *in vivo* relevance is not yet completely defined.

Upon sensing invading viruses, host cells trigger signaling events that ultimately lead to the activation of an innate immune response, characterized by the secretion of interferons (IFNs) and the expression of an array of antiviral factors, including IFN-stimulated genes (ISGs) and inflammatory cytokines [14]. These host responses prevent viral spread and promote the onset of the adaptive immune response. The activation of the innate response typically occurs within infected cells through recognition by innate sensors of viral elements, including viral nucleic acids. These sensors can be cytoplasmic (e.g., retinoic inducible gene-I (RIG-I)-like receptors and nucleotide-binding oligomerization (NOD)-like receptors) or endosomal (e.g., Toll-like receptors, (TLRs)) [15]. Nonetheless, virtually all viruses have evolved mechanisms to evade and/or inhibit these responses within infected cells.

In contrast to these conventional recognition mechanisms within infected cells, in this review we focus on the regulation of innate immunity and viral spread by the responses of uninfected cells in the vicinity of infected cells. The state of naïve cells can be modulated by viral or host components transferred from neighboring infected cells via the release of extracellular vesicles and/or non-infectious viral components. Therefore, the transfer of these different components can promote a host response in the absence of direct infection of cells, with a decreased likelihood that viruses can adapt to avoid these reactions. Here, we present examples of these fascinating, newly-discovered regulatory pathways of viral infection and/or host responses occurring for a broad array of diverse and genetically distant viruses.

## 2. Transmission of Replicating Viral Genomes by Exosomal Transfer

Reports have demonstrated that cells infected by viruses can encapsulate viral RNA within exosomes or exosome-like vesicles as depicted, for example, in the context of infections by the human immunodeficiency virus (HIV), and the hepatitis A and C viruses (HAV and HCV, respectively) [16,17,18,19,20]. Notably, recent studies have demonstrated that HCV infection can be transmitted by HCV RNA-containing exosomes (herein referred to as HCV EXOs), implying an alternative transmission mechanism distinct from infection by canonical virus [21,22]. Despite the potential difficulties in physically separating HCV EXOs from canonical viral particles (e.g., similar buoyant density of both types of vesicles) (Box 1), some studies provided evidence in regards to their discrimination, including an efficient isolation of HCV EXOS from viral particles by immunoprecipitation of CD63, an exosomal marker [21]. Additionally, *in vitro* studies using mutant viral genomes with deletion of viral structural proteins (*i.e.*, the surface glycoproteins E1 and E2, as well as the core protein) revealed that HCV EXOs are secreted even in absence of viral particle production, thus demonstrating that they are structurally distinct from canonical HCV particles [16]. Moreover, HCV EXOs transmit replication-competent viral RNA to naïve hepatic cell lines, even when produced in absence of viral structural proteins, indicating that HCV EXO-mediated cell entry is distinct from conventional infection by virions [23]. In accordance, HCV entry receptors, in concert with E2 glycoprotein, participate either poorly or not at all in the transmission of replication-competent viral genomes by HCV EXOs [21,22]. Altogether, these lines of evidence suggest that the secretion and transmission pathways of either type of carrier (*i.e.*, conventional viral particles or HCV EXOs) are likely distinct from each other, in agreement with the different composition of the two types of vesicles [16,21,22]. Interestingly, a recent study highlights the presence in HCV EXOs of cellular factors known to regulate HCV replication (e.g., miRNA122, HSP90 protein) [21]. It is possible that these elements contribute to the establishment of replication in the recipient cells. The biogenesis of the HCV-EXOs involves cellular factors known to contribute to exosome production, including the ESCRT machinery and Annexin A2 [16]. As mentioned in the introduction, the ESCRT machinery promotes the invagination within the multivesicular bodies (MVBs) of intraluminal vesicles thought to be released as exosomes [7]. HCV RNA colocalized with a subset of ESCRT and exosomal factors in the cells, consistent with the notion that HCV RNA could be incorporated into intracellular vesicles destined to become exosomes [16]. Annexin A2 is a phospholipid-binding protein involved in membrane remodeling and actin cytoskeleton dynamics with a dual cytoplasmic localization, either at the plasma membrane or at the endosome [24]. In particular, Annexin A2 plays a key role in the maturation of endosomes into MVBs [25]. However, the underlying mechanism leading to the biogenesis of HCV-EXOs is still elusive. In addition, it would be of interest to further decipher how exosomes incorporate HCV RNA, and how the pathway of HCV-EXO formation is connected to the production of infectious viral particles [26].

Box 1 **The separation of exosomes, vesicles, and virions.**

Exosomes are the best-studied class of extracellular vesicles, and their role in intercellular communication is a fascinating and nascent field of study. However, as a note of caution, it has been increasingly recognized that exosomes are difficult to define uniquely. Some of the protein markers are shared between exosomes and other extracellular vesicles, such as the plasma membrane-derived microvesicles/ectosomes, including e.g., flotillin and HSP70 [27,28]. Although microvesicles are differentiated on the basis of diameter (e.g., exosomes: 40–100 nm, ectosomes: 100–1000 nm), size-based separation of extracellular vesicles can be approximated given the possible apposition of size ranges for different classes of vesicles [1]. Of note, both exosomes and larger microvesicles are known to contain miRNAs and mRNAs [29,30,31]. While exact biochemical characterization of these vesicular classes remains difficult to clearly define, the function of vesicles in cell-to-cell communication is becoming an important aspect of different fields of cellular biology, including virology and immunology. In the absence of exact methods to define the individual vesicles, here we defer to the authors’ definition of exosomes and other vesicles. The techniques for biochemical separation of exosomes from other classes of microvesicles, and protein markers to characterize the reliability of the separation were recently well-reviewed [32]. Additionally, it should be stressed that some studies, highlighted as such in this review, brought several lines of evidence to define as much as possible the biochemical nature of the vesicles involved in particular cell-to-cell communication.

Enveloped virions often have similar biochemical properties and diameter as compared to exosomes and/or other types of vesicles. Therefore, similar issues apply in differentiating virions from other extracellular vesicles. While discrimination of exosomes from other host-derived vesicles may not be absolutely critical in the study of vesicular cell-to-cell communication, the distinction between virus and host-derived vesicles is essential. Therefore, careful purification of host-derived vesicles from virions (e.g., the absence of viral structural proteins in exosome preparations) and/or the inclusion in studies of mutant virus deficient for the release of extracellular virions are of utmost importance in the elucidation of how host vesicles facilitate cell-to-cell communication during viral infection. In this review, we highlight studies, in which purification of exosomes from virions and/or careful functional analysis of vesicular trafficking apart from virion release have been performed.

A recent publication elegantly showed that HAV, previously defined as a non-enveloped virus, hijacks the exosomal pathway to egress from infected cells as virions that are enveloped by a lipid membrane [17]. Especially, such enveloped HAV virions are formed by enclosing of the non-enveloped viral particles, consisting of the viral genome contained within a capsid, by cell-derived membranes through interactions with ESCRT proteins [17]. Importantly, enveloped HAV readily transmits infectivity, thus challenging our classification of a virus as specifically enveloped *versus* non-enveloped.

Interestingly, both HCV-EXOs and enveloped HAV are resistant to neutralization by antibodies, which target the viral surface glycoproteins [17,22], further demonstrating the distinction of this mode of transmission from canonical pathways of virion infection. Additionally, these findings support the hypothesis that the transfer of infection via exosomes might contribute to viral evasion from the adaptive humoral response, because antibodies targeting viral surface glycoproteins will not, or poorly, block this type of transmission.

## 3. pDC Activation by Vesicle-Mediated Transfer of Viral RNA

The plasmacytoid dendritic cell (pDC) is an immune cell type known to play a crucial role in the activation of the innate response against viral infection, mainly via the recognition of viral nucleic acids. This is accomplished primarily by TLR7 and TLR9, whose ligands are single-stranded RNA and unmethylated CpG-containing DNA, respectively [33]. Recognition of these signals leads to pDC activation, characterized by the production of immune mediators, including type I IFN. These molecules subsequently activate the production of an array of antiviral factors.

### 3.1. pDC Activation by Exosomal Transfer

**Figure 1:**
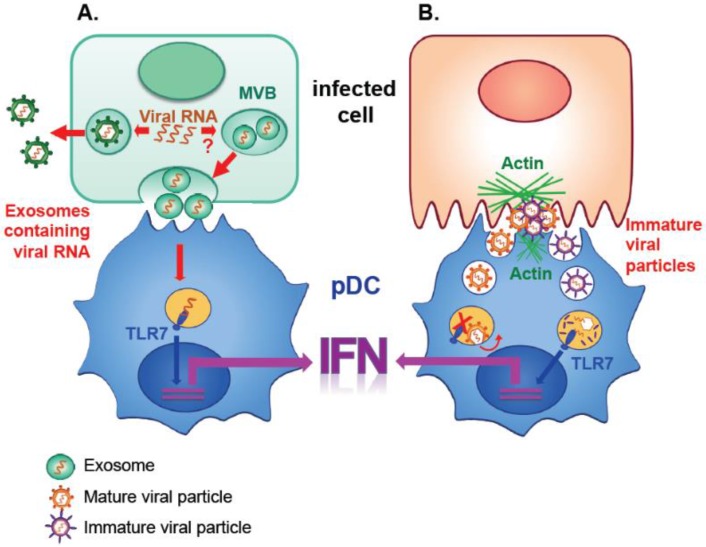
**Models of pDC activation by viral components released by infected cells independently of productive infection.** (**A**) The activation of pDC IFN response triggered by exosomes produced by infected cells, such as e.g., upon HCV, LCMV infection. In parallel to infectious viral production, the viral RNAs are packaged in exosomes within multivesicular bodies (MVBs) in infected cells and subsequently transmitted to a pDC engaged in direct physical contact with an infected cell. The viral RNA is then recognized by TLR7 in pDC, leading to the production of type I IFN; (**B**) The activation of pDC IFN response triggered by immature particles produced by dengue virus (DENV) infected cells. pDCs and infected cells establish cell-to-cell contacts. The viral particles form clusters at the interface. The actin network is also polarized toward this contact and likely acts as a structural platform for transmission of viral components, including viral RNAs, to pDCs. Such transmission also requires the structural viral proteins. Mature DENV particles, which are fusion-competent (orange), poorly activate the pDCs, likely because they could escape by membrane fusion from the recognition by TLR7, which is localized in the endo-lysosomal compartment where the membrane fusion occurs. In contrast, immature DENV particles, which are non-fusogenic (purple), could be retained in this compartment, leading to the release of viral RNA within the endo-lysosome and recognition by TLR7, hereby resulting in a robust production of type I IFN by pDCs.

Interestingly, as exemplified in the context of HCV, the transfer of viral RNAs by exosomes has a dual role in the progression of infection, and likely also pathogenesis, through transmission of both infection (to the target cells, the hepatocytes) and an activating signal (to innate immune cells). These events lead to both viral spread and the establishment of an antiviral state, depending on the target cell type [16,17,21,22,34,35]. Especially, recent *in vitro* studies have demonstrated that exosomes produced by infected cells play a key function in the activation of the innate immune response by pDCs, including the production of type I IFN [16,34] (Figure 1). Importantly, this newly-discovered exosome-mediated activation of innate immunity is functionally conserved between evolutionarily distant viruses, since it was reported for both a positive-sense single-stranded RNA virus of the *Flaviviridae* family *i.e.*, HCV [16] and a negative-sense single-stranded RNA virus of the *Arenaviridae* family, *i.e.*, the lymphocytic choriomeningitis virus (LCMV) [34]. Additionally, the above-mentioned enveloped form of HAV, produced via a cellular pathway resembling the exosome biogenesis route, also transfers a stimulating signal to pDCs [17,35]. Consistently, ALIX (also known as programmed cell death 6 interacting protein, PDCD6IP), an ESCRT factor and exosome marker [36], co-sediments with the pDC activating signal-containing fraction and is present in enveloped HAV [35]. Nonetheless, some other structural properties of the enveloped HAV are distinct from the expected composition of exosomes, such as the absence in enveloped HAV of acetylcholinesterase, another exosome marker [35]. Therefore, despite sharing some characteristics and markers with exosomes, the exact nature of enveloped HAV remains enigmatic.

Interestingly, pDCs may preferentially respond to viral RNA transferred by exosomes rather than by conventional viral particles. In accordance with this hypothesis, while HCV-EXOs induce a strong pDC IFN response [16], on the contrary, infectious HCV particles block pDC responsiveness to TLR7-induced signaling [37]. In particular, the binding of the HCV E2 glycoprotein to a cell surface-expressed lectin, BDCA-2, is responsible for the negative regulation of the pDC IFN response [37]. This type of inhibition by viral particles or subviral particles has been also reported for other viruses, including human papillomavirus and hepatitis B virus (HBV) [38,39]. Therefore, exosomes containing immunostimulatory signals may act as heightened carriers for an antiviral pDC response.

Altogether, current evidence highlights separate modalities in the transmission of exosomes containing viral RNAs to recipient cells, including pDCs, in comparison to canonical infectious virions. How these exosomes or exosome-like vesicles are captured, recognized, and then internalized to deliver their cargo to the appropriate compartment of the recipient cells to eventually modulate their function is still enigmatic. Additionally, to the best of our knowledge, the activation of innate immunity through the recognition of exosome-transferred viral genome has only been described in the context of pDCs. Nonetheless, it is indeed likely that such cell-to-cell transmission of immunostimulatory signals also activates other cell types and/or innate immune responses, as further discussed in Section 4.

### 3.2. pDC Activation Independently of Exosomal Transfer

In sharp contrast to the viral envelope protein-independent pDC response to cells infected by either HCV, LCMV or classical swine fever virus (CSFV) [34,40,41], pDC activation by dengue virus (DENV), a flavivirus, is induced by viral envelope protein-dependent secretion and transfer of viral RNA [42]. Consistently, structural viral components cluster at the interface between DENV infected cells and pDCs [42]. This clustering at the contact leads to cell-to-cell transmission and endocytosis by pDCs of the viral RNA encapsulated within virions [42].

For many viruses, newly formed particles undergo maturation by cleavage of the viral surface proteins in the secretory pathway [43]. This processing step renders the virus infectious [43]. In the context of DENV, premembrane (prM) surface viral protein is cleaved into M protein. However, prM cleavage site is suboptimal, leading to the secretion of about 30%–40% immature, prM-bearing particles [44,45,46,47,48,49]. This evolutionarily-conserved suboptimal cleavage site may positively contribute to viral infection by usurping the humoral immune response for its cell entry [44,46,50,51,52]. Consistently, indirect evidence supports the *in vivo* existence of uncleaved prM-containing viral particles. For example, antibodies against prM form a major component of the serological response to DENV infection [53,54,55]. Interestingly, the level of pDC activation by DENV infected cells depends on the degree of maturation of viral particles produced by infected cells [42]. Indeed, cells producing more immature particles are more potent at activating pDCs as compared to those producing mature particles [42]. The IFN response by pDCs to DENV infected cells involves the RNA sensor TLR7 localized in the endo-lysosomal compartment [42]. The membrane fusion of DENV is known to be triggered by acidic pH, implying that this event also occurs within the endo-lysosome [56]. Therefore, this observation led to the proposition of a working model in which immature virions (*i.e.*, incompetent for membrane fusion) are likely retained in the endo-lysosome compartment, resulting in a potent TLR7-mediated activation of pDC IFN production. In sharp contrast, cells producing more mature particles are poor inducers of pDC IFN production, likely because the fusion-competent mature particles release viral RNA into the cytosol by membrane fusion. Therefore, these mature particles would evade TLR7 recognition (Figure 1). Future study is needed to formally validate this working model. Interestingly, this newly-discovered concept may have broad importance for the many viruses that, like DENV, can prevent activation of the pathogen-sensing machinery within infected cells and can release non-infectious particles containing uncleaved glycoproteins, such as influenza virus [57,58] and WNV [53,59].

### 3.3. Common Features of pDC Activation

Interestingly, similarities are observed in the mechanism of pDC activation triggered by vesicular transfer of immunostimulatory viral RNA, either by exosomes or non-exosomal pathways. In particular, multiple lines of evidence suggested that pDC activation requires the establishment of cell-to-cell contacts between pDCs and infected cells. This key feature of pDC activation is shared by evolutionary distant RNA viruses, including members of the *Flaviviridae*, *Arenaviridae*, *Retroviridae* and *Picornaviridae* families [16,34,35,40,41,42,60,61] and potentially DNA viruses, such as the herpes simplex virus (HSV) [62]. Indirect experimental evidence suggests that infected cells likely secrete immunostimulatory vesicles into the culture supernatant at concentrations below the stimulatory threshold required for pDC response, while this level could be reached in the intercellular space during cell-to-cell contact [16,42]. In accordance with this hypothesis, a more detailed analysis of cell-to-cell contacts between pDCs and infected cells, in the context of DENV infection, revealed that structural viral components cluster at such contacts. In addition, the actin cytoskeleton accumulates at the site of contact between a pDC and an infected cell, favoring the establishment of cell-cell interactions [42]. These observations suggest that the actin network acts as a structural platform for the transmission of the activating signal to pDCs [42]. Yellow fever virus (YFV), another flavivirus, also induces a strong pDC IFN response by cell-to-cell contacts, as opposed to a very weak activation by cell-free virus [60]. This emerging model for a cell-to-cell contact-dependent mechanism of pDC activation by infected cells argues for the existence of a localized pDC response at the site of infection. Importantly, several *in vivo* studies of HCV infected patients and HAV infected chimpanzees demonstrate that pDCs are readily recruited to the infected liver [35,63,64]. Additionally, this recruitment of pDCs temporally coincides with the production of antiviral factors, as shown in the context of HAV infected chimpanzees [35]; hence, further supporting the relevance of a localized pDC response occurring during viral infection.

For many viruses, the pDC response to infected cells does not require productive infection of the pDCs, since the associated amplification of the viral genome and/or expression of viral proteins is not detectable in activated pDCs. For instance, lack of viral replication in pDCs has been demonstrated in the context of infection by the *flaviviridae* members DENV, WNV [42], and HCV [40], as well as viruses from other distinct families, including HAV [35] and influenza virus [65]. Consistently, for these different viruses, viral replication in pDCs is not required for their activation, congruent with the result that UV-inactivated virions are still capable of activating IFN production by pDCs [62,66]. pDCs are robust producers of type I IFN, in part due to the constitutive expression of IRF7, a transcriptional factor downstream of the TLR7-induced signaling [67]. Therefore, it is conceivable that this rapid IFN response renders them refractory to infection by most viruses.

Altogether, these observations emphasize many common features of pDC activation by RNA viruses. Nonetheless, some features of pDC activation are restricted to particular viruses. One might speculate that virus-specific features of pDC activation (*i.e.*, the type of vesicle, which transfers the immunostimulatory RNA to pDCs) could be directed and/or result from the distinct biology and life cycle of each viral family or genus. For instance, the subcellular localization of the viral RNA in infected cells is different for various viruses, depending on the cellular machinery used for viral replication and/or assembly. Such specificities to a particular virus could cause viral RNAs to be incorporated within different types of non-conventional carriers, e.g., exosomes *versus* immature virions. Alternatively, the virus-specific characteristics of the pDC IFN response may result from differential viral inhibitory and/or escape mechanisms, e.g., inhibition of the egress of the pDC activating signal or blockade of pDC responsiveness. Conversely, the features of pDC activation that are conserved across viral families are likely dictated by the characteristics of the signaling activation pathway in pDCs. In particular, the localization of the immune sensor TLR7 in the endo-lysosomal compartment [67] implies internalization of the immunostimulatory signal. In accordance, pDC IFN production does not require productive infection by the viral particles since the signaling can be activated by recognition of incoming viral RNA during internalization. How exactly the viral genomes present in internalized vesicles are exposed and recognized by immune sensors is still enigmatic. Nonetheless, it is conceivable that proteases and lipases present in the endo-lysosomal compartment contribute to the digestion and exposure of the nucleic acid from the carrier to the sensor. Importantly, as yet unrecognized factors expressed at the cell surface of pDCs, may be pivotal i) for the establishment of cell-to-cell contacts with various types of infected cells, and/or ii) for the remodeling/polarization of cellular factors at the contact site to favor the transmission of the activating signal, such as the intracellular actin cytoskeleton [42].

## 4. Immune Activation by Cell-to-Cell Transfer of Viral Elements from Infected Cells

Virally-derived components released by infected cells can also induce innate immune responses in neighboring uninfected cells, including cell types other than pDCs. These responses are likewise independent of the transfer of conventional infectious virus and/or establishment of infection. Such non-canonical communication to uninfected cells, playing both immune and non-immune functions, likely also contributes to the progression of viral infection and pathogenesis. Although these alternative transmission mechanisms can be diverse in nature, we focus here on a subset of pathways recently described in the context of innate immune activation by transfer of viral RNA species from HCV infected cells. This represents a very active recent area of investigation.

Recent reports illustrate the sensing of infected cells by an immune cell type known to produce large amounts of type III IFNs, the BDCA-3^+^ or CD141^+^ myeloid DC, also called mDC2. Type III IFNs lead to the expression of an array of antiviral factors, including IFN-stimulated genes (ISGs). BDCA-3^+^ DC appears to be the major producer of type III IFN among peripheral blood mononuclear cell populations in response to HCV infected cells [64,68]. The BDCA-3^+^ DCs sense infected cells via recognition of the viral RNA by TLR3 [64]. Although still debated, this response might require physical cell-to-cell contacts [64]. Importantly, virus production is not required to trigger type III IFN production by BDCA3^+^ cells, because cells that replicate a mutated form of HCV genome that does not produce viral particles similarly trigger this response [64]. Altogether these observations suggest that activation of type III IFN production by BDCA-3^+^ cells shares similarities with the previously identified activation pathway of IFN production by pDCs (*i.e.*, dependent on cell-to-cell contact but not on infectious virus production). Interestingly, additional evidence suggests the existence of regulatory cross-talk between different signaling pathways triggered by HCV infected cells. For example, pDC-derived type I IFNs potentiate the production of type III IFNs by BDCA-3^+^ cells [64].

Another recent study demonstrated an alternative sensing mechanism of infected cells by uninfected adjacent cells, including hepatocytes, also via the sensor TLR3 [69]. This sensing leads to the induction of an antiviral state, which restricts HCV replication primarily via the production of IFN-β, a type I IFN [69]. Activation of this antiviral response involves the recognition of secreted HCV double-stranded (ds)RNA by TLR3 [69]. This RNA specie released by HCV-infected cells is likely the annealed form of the positive strand of the viral RNA genome and the replication intermediate of negative polarity [69]. However, it is unclear whether dsRNA is released from HCV infected cells as a free-RNA specie or incorporated within vesicles. Importantly, the surface-exposed class A macrophage scavenger receptor (MSR-1) is required for the binding and transfer of the extracellular dsRNA to endosome-localized TLR3 [70,71]. This newly discovered aspect of TLR3-induced signaling implies a non-canonical transmission of dsRNA to uninfected neighboring cells independent of infectious viral particles. How the dsRNA intermediate, thought to be mainly localized at the replication site (for HCV, the ER-derived membranes) [72], is targeted to the appropriate cellular compartment for its egress and release from infected cells is still enigmatic. Interestingly, similarly to the above-mentioned activation of the innate response by pDCs, MSR1/TLR3-dependent sensing requires close proximity with infected hepatocytes and likely direct cell-to-cell contact [69]. Such signaling might have broader importance for other viruses. In particular, vesicular stomatitis virus (VSV) infection was demonstrated to activate TLR3 via another member of the class A scavenger receptors *in vivo* [70]. Of equal interest will be to determine whether MSR1/TLR3-dependent sensing of dsRNA might also contribute to other responses, such as the activation of Kupffer cells known to express MSR1.

Along this line, another original mechanism of activation of the innate immune response, in this case the inflammasome, by HCV has recently been highlighted [73]. Inflammasomes are multiprotein complexes formed upon sensing of virus infection, which lead to the secretion of proinflammatory cytokines, IL-1β and IL-18. The induction of this response has been linked to the liver damage associated with chronic inflammation during HCV infection [73,74]. *In vitro* studies revealed that HCV induces the inflammasome and concomitant IL-1β secretion by Kupffer cells and monocyte-derived macrophages. Since myeloid cells are thought to be refractory to HCV infection, phagocytic uptake of HCV particles and/or other viral components is likely sufficient for the recognition of viral RNA by the endo-lysosome-localized TLR7, leading to the induction of the inflammasome [73]. Accordingly, these events occur in absence of productive infection [73]. It would be of interest to determine whether carriers of HCV RNA other than conventional infectious virions (e.g., exosomes) can trigger this response and whether this activation mechanism holds true for other viruses. For example, HIV-mediated inflammasome activation is similarly independent of productive infection and dependent on endocytic uptake of viruses, leading to activation of the inflammasome in monocytes via TLR8 (another ssRNA sensor) and not TLR7 [75]. Nonetheless, inflammasome activation in the absence of productive infection is not consistent across different viral infections. For instance, in the context of influenza virus infection of macrophages, activation of the inflammasome via TLR7 requires productive infection [76]. Therefore, viruses may generally signal through TLRs to activate the inflammasome in myeloid cells. However, the specific TLR used and the requirement for productive infection in the activation of myeloid cells depends on the particular biology of each virus.

This selected subset of recent findings on the recognition mechanisms of infected cells, uniquely regarding HCV, illustrates how varied mechanisms of non-canonical cell-to-cell transfer of immunostimulatory signals lead to an innate immune response. Interestingly, these non-canonical pathways of viral recognition share a common theme: productive infection is not required to induce innate immunity. These innate immune responses involve recognition of viral RNA by endosomal/endo-lysosomal-localized sensors (*i.e.*, TLR3 and TLR7 [15]). Thus, similarly to pDC activation by non-canonical vesicles (see Section 3.3), it is possible that incoming viral RNAs are recognized by these sensors during internalization and, thus, without the need of productive infection. Nevertheless, the underlying mechanism of activation varies by the involvement of viral particles or other carriers, their entry pathway, and the type of response induced (*i.e.*, the specific recognition pathway and cytokine(s) produced). Further, the diversity of RNA transfer and recognition mechanisms may be affected by the particular biology of each cell type. While we have chosen HCV as a representative virus for recent studies, such manifold modes of innate immunity induction by the non-canonical transfer of viral components, in the absence of productive infection, are likely to generally extend to other viruses.

## 5. Transfer of Regulatory Components by Exosomes

### 5.1. Virally-Derived MicroRNAs

As discussed above, exosomes can transfer RNA species derived from infected cells to neighboring cells, including full-length genomes or dsRNA (see previous Sections). An additional type of RNA, the microRNA (miRNA) is well-known to be transferred from cell to cell by extracellular vesicles, especially by exosomes [77,78]. This is likely in part because the miRNA machinery is localized within MBVs and, thus, intersects with the exosome biogenesis pathway [79,80]. Neighboring cells may take up exosome-resident miRNA by fusion of the exosome with the plasma membrane or endocytic membrane [81,82]. However, this process is presently poorly understood. Here, we present several examples illustrating the potential effects of exosomal-transferred miRNAs on neighboring cells and/or systemic responses in the host during infection. In contrast to previously-discussed exosome-mediated viral genome transfer, transmission of miRNAs can have more widely-ranging effects than only, or primarily, induction of an antiviral response, given the ability of miRNAs to target mRNAs involved in sundry cellular pathways. This exciting and nascent research area in virology is mainly supported by *in vitro* studies. The *in vivo* importance of this type of communication is still an uncharted and challenging domain of investigation.

**Figure 2:**
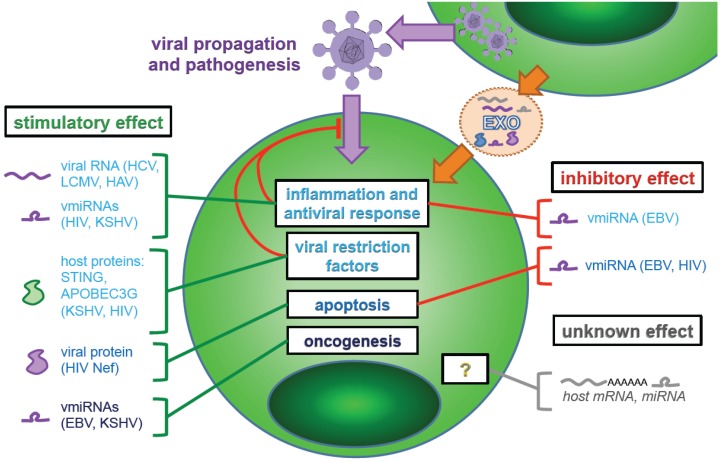
**Exosome-mediated transfer of host and viral components from infected to neighboring cells.** Upon infection, the contents of exosomes produced by infected cells considerably change, in regards to host proteins, RNAs, and incorporation of micro RNAs derived from the host (miRNA) or from the viral genome (viral RNA, vmiRNA). The host- and virally-derived species present in exosomes are transferred to neighboring cells, where they can either activate or inhibit an array of different host signaling pathways. These exosome-mediated communications have a broad effect on the functionality of cells in the vicinity of infected cells, including on the cell types that are refractory to productive viral infection. The scheme displays a selection of different species transferred by exosomes known to affect the course of viral infection and/or pathogenesis by targeting inflammatory, antiviral (via viral restriction factors), apoptotic, and oncogenic pathways. Significant changes occur in the contents of host-derived mRNA and miRNAs that are encapsulated in exosomes upon viral infections yet, to date, the effect of these different RNA species on neighboring cells during a viral infection is incompletely understood.

Viral infection induces important changes in the exosome molecular signature, including the incorporation of miRNAs derived from the viral genome (referred to herein as vmiRNAs). Exosomal transfer of vmiRNAs was first described in 2010 [83,84,85] (Figure 2 and Table 1). Several recently published examples illustrate that vmiRNA can both positively or negatively regulate inflammatory responses depending on the type of viral infection. For instance, Epstein-Barr virus (EBV)-infected cells release vmiRNAs in exosomes, leading to signaling changes in neighboring cells [83,84], notably the repression of immune-related genes [84,86], which may facilitate viral persistence. Importantly, an EBV-derived vmiRNA transferred in exosomes (miR-BART15) targets the *NLRP3* inflammasome gene and as a result, decreases the secretion of the proinflammatory cytokine IL-1β by uninfected cells [86]. Furthermore, EBV-infected B cells can transfer vmiRNAs to dendritic cells (DCs) and T cells [87,88], cell types that are not normally infected by EBV. This would therefore allow EBV to control inflammatory signaling in these cell types, in the absence of direct regulation by productive infection. Other EBV-derived vmiRNAs (miR-BART-1, miR-BART-2, miR-BART-3) similarly target host proteins involved in inflammation and are found in exosomes derived from EBV-infected cells [89,90,91,92,93,94] (Table 1), yet how they functionally regulate the neighboring cell function remains to be defined. Therefore, together these examples illustrate the existence of parallel regulation of a type of host response, inflammation, by different exosome-transferred vmiRNAs that are derived from a given viral genome (Table 1).

**Table 1:** Examples of regulatory functions of viral miRNAs secreted in exosomes. N.D.; non-demonstrated, *i.e.*, the effects of these vmiRNAs—transferred by exosomes—on neighboring cells have not yet been definitively demonstrated. N.P.: not performed; EM: electron microscopy.

Virus	Name of Viral miRNA(s)	Source of Exosome-Resident vmiRNAs	vmiRNA Effect	vmiRNA Target(s)	Exosome/Virion Separation Validation	Exosome/Other Microvesicle Separation Validation
EBV	miR-BART15	Namalwa B cell line	Anti-inflammatory	NLRP3 mRNA [86]	Cells do not produce virions	N.P.
		AGS gastric cancer cell line	Anti-apoptotic	BRUCE mRNA [95]	N.P.	CD9, CD81 western blot
	BHRF-1-3	LCL lymphoblastoid B cell line	Anti-inflammatory	CXCL11 mRNA [84]	EM; no large RNAs in exosomes	EM; CD63 western blot
	miR-BART3	NPC nasopharyngeal carcinoma cell line	Anti-inflammatory (N.D.)	IPO7 mRNA (IL-6 inducer) [92,93]	N.P.	Exosome Co-IP with CD9, MHC II
	miR-BART1	NPC nasopharyngeal carcinoma cell line	Immune evasion (N.D)	LMP1 mRNA (viral membrane protein) [93,96]	N.P.	Exosome Co-IP with CD9, MHC II
	miR-BART2	NPC nasopharyngeal carcinoma cell line	Immune evasion (N.D)	MICB mRNA [89,90,93]	N.P.	Exosome Co-IP with CD9, MHC II
		NPC nasopharyngeal carcinoma cell line	Latency promotion (N.D)	BALF5 mRNA (viral polymerase) [93,94]	N.P.	Exosome Co-IP with CD9, MHC II
KSHV	miR-K12-(4-5p, 4-3p, 5, 6-5p, 10a, 11)	Patient serum, pleural fluid	Pro-inflammatory, cell migration	Targets unknown (increased IL-6 expression) [97]	No viral DNA (QPCR); no viral protein (Western blotting); EM	Exosome Co-IP with CD63; EM; Flotillin, HSP90, CD9, CD63 western blot
HIV	miR-TAR	Jurkat lymphoblastoid T cell line	Anti-apoptotic, increased susceptibility to HIV-1 infection	Bim/CDK9 mRNA [98]	No viral DNA (QPCR)	EM; CD45, HSP70, β-actin, Alix, CD63 western blot
	vmiR88, vmiR99	Macrophage-differentiated THP-1 promonocytic cell line	Pro-inflammatory	Direct TLR8 stimulation (TNF-α release) [99]	N.P.	CD63 western blot

Conversely, vmiRNAs derived from other viruses can have a proinflammatory effect or more complex effects on the inflammatory responses (Figure 2 and Table 1). For example, studies of tumor samples from patients infected by Kaposi’s sarcoma-associated herpesvirus (KSHV) demonstrated that vmiRNAs transferred via exosomes enhance pro-inflammatory IL-6 secretion [97]. Interestingly, KSHV is also known to separately induce IL-6 signaling through a viral mimic protein of IL-6 [100,101]. The viral mimic protein and exosomal export of vmiRNA likely lead to complementary activation of the IL-6 pathway, both within infected cells and in neighboring cells, respectively. Another example of pro-inflammatory function of vmiRNAs transferred by exosomes was highlighted in a study of HIV-infected patients [99]. Exosomes containing HIV-derived vmiRNAs (*i.e.*, vmiR88 and vmiR99) lead to secretion of TNF-α, a proinflammatory cytokine, by macrophages [99]. Interestingly, the dependence on TLR8 of this response implies that these vmiRNAs likely act as RNA species recognized by the ssRNA sensor TLR8 [102], rather than by regulating host mRNA expression [99]. Such an activation mechanism may play a role in the long-term chronic inflammation by creating a circulating pro-inflammatory vesicular signal [99]. Altogether these selected examples focused on the regulation of inflammation point out that exosomal transfer of vmiRNAs can systemically signal to uninfected cells and, hence, may affect correlates of chronic viral diseases.

In addition to the regulation of the inflammatory status, vmiRNAs transferred by exosomes can also have supplementary effects on the disease outcome by contributing to virus induced-oncogenesis. For example, IL-6 secretion, triggered by KSHV vmiRNAs as mentioned above, can lead to the expression of the pro-inflammatory chemokine CCL2, which has pro-oncogenic effects, *i.e.*, stimulation of angiogenesis and endothelial cell migration [97,103,104]. Moreover, these inflammatory signals are known to induce the lytic phase of replication in KSHV, which also facilitates tumorigenesis [105]. Therefore, the KSHV-derived vmiRNAs transferred in exosomes may have distinct regulatory functions that potentially promote a switch in the viral life cycle and tumor development. Along the same line, gene ontology analysis revealed that, in addition to pro-inflammatory pathways, EBV-derived vmiRNAs mainly target factors of various pathways linked to cancer development [106]. Especially, EBV vmiRNA, miR-BART15, is transferred by exosomes and induces apoptosis in neighboring cells by targeting an anti-apoptotic regulator [95]. In this study, the authors speculated that this may facilitate tumor growth in EBV-infected patients by eliciting apoptosis in neighboring immune cells. As opposed to this positive regulation of apoptosis, a vmiRNA derived from the HIV-1 transactivation-response element (TAR) RNA, which is secreted in exosomes from infected lymphocytes and patient sera, down-modulates apoptosis by inhibiting pro-apoptotic factors [98]. Interestingly, the TAR-derived vmiRNA also increases the susceptibility of lymphocytic/monocytic cell lines to HIV-1 infection through an as-yet unknown mechanism [98]. Given the ability of vmiRNAs to target many cellular pathways, exosome-transferred vmiRNAs may influence multiple parameters regulating the course of disease, as exemplified here on immune response, along with apoptosis and oncogenesis. An emergent concept is that vmiRNAs might regulate in a complex network the viral infection and disease progression.

### 5.2. Transfer of Host-Derived miRNAs, mRNAs and Proteins by Exosomes

In addition to the virally-derived RNA species, host-derived components incorporated into exosomes can also regulate the host response to viruses and, thereby, infection progression and/or pathogenesis (Figure 2 and Table 1). Multiple studies have highlighted significant virally-induced changes in the content of host proteins present in exosomes [107]. For example, the exosome protein content changes in HIV-infected T cell lines [108], gammesherpesvirus (KSHV, EBV)-infected B cell lines [109], and HBV-infected hepatocyte cell lines [110] (*i.e.*, altered exosome incorporation ranged from a low of 6.2% of proteins in HIV-infected cells to ~21% of proteins in gammaherpesvirus-infected cells). Among the many exosome-resident proteins identified in these studies, some are associated with immune responses, shedding light on how these global changes in exosome-resident host proteins might modulate the course of viral infections.

In the context of herpes simplex virus-1 infection (HSV-1), Kalamvoki *et al.* [111], elegantly demonstrated that infected cells secrete exosomes containing notably the host stimulator of interferon genes (STING) protein. STING is an innate immune adaptor and a potent activator of the IFN pathway [112,113]. In this study the exosomes were clearly delineated from virions and other microvesicles by using CD9 immunoprecipitation, and by demonstrating an efficient vesicular transfer of STING to neighboring cells even in the presence of HSV-1 neutralizing antibodies [111]. Moreover, the HSV-1 protein ICP0 stabilizes STING expression by preventing its degradation [112] and facilitates its export from cells by exosomes [112]. This export is induced upon HSV-1 infection, thereby making surrounding cells resistant to infection, which is a somewhat counterintuitive viral strategy [112]. As proposed by the authors, virally-mediated exosome export of the STING protein may actually be key in favoring viral dissemination to new hosts [112]. HSV-1, following initial infection, normally becomes latent within the neuronal compartment. Reactivation and shedding of the virus requires anterograde transport down the neuron to the exterior of the body [114]. Infection of surrounding neurons would not favor inter-host virus dissemination, since hosts with damaged central nervous systems are less gregarious [115]. Therefore, preventing infection in surrounding neurons via transfer of STING would thus favor dissemination of the virus to other hosts. This is a compelling hypothesis for how a virus may hijack, via an unexpected, original way, the exosomal communication machinery to its advantage.

Conversely, other host antiviral factors transferred by exosomes modulate the course of an infection in the favor of the host. For example, interferon-induced transmembrane protein 3 (IFITM3) can be transferred from cell to cell by exosomes, thereby preventing DENV cell entry [32,116] (discussed in [32]). Along the same lines, Khatua *et al.*, demonstrated that exosomes derived from a T lymphocyte cell line, chronically infected with HIV, induced an antiviral state in treated cells [117]. Notably, a single protein was responsible for nearly all of the exosomal anti-HIV activity, Apolipoprotein B mRNA-editing, enzyme-catalytic, polypeptide-like 3G (APOBEC3G), an HIV restriction factor. However, this antiviral effect does not require the cytidine deaminase activity of the APOBEC3G protein, which typically acts to lethally mutagenize viral genomes during reverse transcription [118,119]. Instead, this effect is likely mediated by the retroviral genome sequestering ability of APOBEC3G [120,121]. In this study, exosomes were separated by CD63 immunoprecipitation and APOBEC3G cosedimented with a number of other exosomal markers, demonstrating a clear isolation of exosomes from other microvesicles. In the context of HBV infection, Li *et al.* [122], recently demonstrated that exosomes produced by uninfected, IFN-α-treated liver nonparenchymal cells can transfer IFN-α-induced antiviral activity between cells. In this study, exosomes were unambiguously separated from other microvesicles by CD63 immunoprecipitation. Specifically, IFN-α treatment greatly changes the content of exosome-associated host-derived miRNAs, mRNAs, and proteins. Similarly to the anti-HIV activity of exosomes, a large part of the exosomally-transferred anti-HBV activity is mediated by the APOBEC3G protein, which also has an antiviral activity against HBV. Although at first, interferon-stimulated genes (ISGs) are thought to play cell-intrinsic functions, these selected examples demonstrate how two ISGs (*i.e.*, STING and APOBEC3G) can induce an antiviral state via exosomal-transfer to neighboring cells, in the absence of transfer of IFN.

Additionally, the profile of host-derived miRNAs encapsulated into exosomes is modified upon viral infection [97,123], thus altering intercellular communication. The exosome-miRNA profile can change considerably, even between viral strains, or according to expression of specific viral proteins [97,123,124]. For instance, the expression of exosome-associated miRNAs is different when comparing two influenza virus strains (H1N1 and H7N7) [123]. In the case of the human papilloma virus (HPV), expression of viral proteins (E6/E7) was sufficient to significantly change the quantity of several types of exosome-resident miRNAs [124]. Differential effects on viral spread or outcome of infection may be observed depending on the targets of the miRNA(s) involved and the targets’ positive or negative regulatory function on the immune response.

Another area of interest, although also still in its infancy, is how the composition of host-derived mRNAs changes in exosomes following viral infection. Exosome-transferred mRNA can be translated in target cells [78]. Therefore, analogous to the above-mentioned regulation by exosome-transferred miRNAs, the transmission of host mRNAs may alter the signaling pathways in neighboring uninfected cells. As an example to illustrate this concept, a recent report demonstrated that human semen contains exosomes with anti-HIV activity, and that these exosomes contain a number of host-derived mRNAs [125]. These mRNAs may be responsible for the exosomes’ anti-HIV function, as these mRNAs include host restriction factors against HIV, such as the *APOBEC3* family members and *Bone marrow stromal antigen 2* (*BST-2*). Notably, addition of human semen exosomes to murine vaginal cells led to the transfer of human APOBEC3F/G mRNA into the murine cells [126]; yet whether this mRNA is expressed and has an antiviral function is still unknown.

Altogether, these recent studies demonstrate that exosomal transfer of host- and virus-derived RNA species and proteins exerts important and diverse functions in viral infections. Open questions include how the vmiRNAs are incorporated and how specific host-derived miRNAs, proteins and, potentially, mRNAs are differentially sorted into exosomes upon infection [77]. In this regard, it is still unclear whether viruses actively modulate the content of exosomes, or whether changes in their composition simply result from intracellular expression modulations during infection. For example, certain of the miRNAs upregulated intracellularly by HPV E6/E7 expression, were also incorporated into exosomes in higher amounts [124]. An additional possibility is that, since exosomes are derived from the MVBs where miRNA processing also takes place, perhaps viral infection perturbs the subcellular localization and/or recruitment of RNA species and proteins in this compartment. Alternatively, viral proteins may actively modulate the miRNA or MVB machinery in order to specifically incorporate certain miRNAs (either host- or virally-derived) into exosomes. An additional open topic of study is the elucidation of the specific candidate host-derived miRNA(s), mRNA(s), or protein(s) responsible for significant changes in the infection progression and/or pathogenesis. This is a very challenging research area because up to hundreds of these species are incorporated into exosomes during infection.

## 6. Conclusions and Perspectives

Innate immunity represents the first line of defense against viral infections, implying that viral persistence requires the virus to evade and/or inhibit this host response. Not surprisingly, virtually all viruses act to subvert innate immune responses within infected cells. As discussed in this review, aside from the activation by infectious viral particles, recent work illustrates the existence of alternative sensing mechanisms of infected cells by neighboring cells that likely constitutes an important aspect of innate immunity. Notably, these surveillance mechanisms are mostly independent of the productive infection of the cells. As a consequence, no viral protein is newly produced in the target cells, implying that the innate signaling pathways are unlikely to be opposed by viral inhibitory processes, or if so, only by the incoming structural viral component(s). Additionally, in productively infected cells, viral components, such as viral nucleic acids, are usually protected from innate sensor recognition via their inclusion in virus-induced intracellular structures, including the viral replication sites. In contrast, in the absence of actively replicating virus and the attendant protective structures, these activating signals are more likely to be exposed to recognition by innate immune receptors in non-productively infected cells. Therefore, it is tempting to speculate that such pathways impose an extremely high barrier to viral adaptation, because the only possible inhibitory mechanism would be borne by incoming viral or host components transmitted along with the stimulatory signal. This latter possibility is exemplified in the context of CSFV infection, as the viral structural protein Erns inhibits pDC activation via its ribonuclease activity [41]. We cannot exclude that these alternative sensing mechanisms of infected cells by the host are an opportunistic response to side-products generated by infected cells. Alternatively, these specific pathways to recognize non-infectious components might have evolved in the host to bypass virus-mediated inhibition of immune recognition within productively infected cells. Consistently, non-conventional and/or non-infectious particles induce a more potent innate immune response than canonical infectious viral particles, as illustrated in the context of pDC activation by immature virions [42].

Interestingly, cell-to-cell contact is emerging as a common requirement for the activation of different types of innate immune responses. One might speculate that such a conserved feature could promote a highly localized response, instead of a systemic immune response and attendant damages, such as elevated levels of cytokines and inflammatory processes that are detrimental to the host. Those characteristics might be especially important for robust and rapid responder immune cell types, such as pDCs. Especially, short-range sensing by pDC of infected cells could limit the infection to a restricted area via a potent and localized antiviral response. Much remains to be elucidated on this highly appealing, newly described aspect of innate immunity and viral infection. Particularly, how do innate immune cells establish cell-to-cell contact with infected cells? How is the information transferred from cell-to-cell? How might both the virus and the host use, inhibit or usurp these transmission mechanisms to regulate infection toward their mutually-exclusive benefit? We expect that the answers to these questions will reflect a more systems-based approach to infection and immunity, where information transferred between infected and naïve cells obviates simplified interactions between one virus and one host cell.
